# Thermal degradation of sucralose: a combination of analytical methods to determine stability and chlorinated byproducts

**DOI:** 10.1038/srep09598

**Published:** 2015-04-15

**Authors:** Diogo N. de Oliveira, Maico de Menezes, Rodrigo R. Catharino

**Affiliations:** 1 INNOVARE Biomarkers Laboratory, School of Pharmaceutical Sciences, University of Campinas, Campinas, São Paulo, Brazil, 13083-877

## Abstract

In the late years, much attention has been brought to the scientific community regarding the safety of sucralose and its industrial applications. Although it is the most used artificial sweetener in foods and pharmaceuticals, many questions still arise on its potential to form chlorinated byproducts in high temperatures, as demonstrated by several recent studies. In the present contribution, we use a combination of differential scanning calorimetry and thermogravimetric analysis coupled with infrared spectroscopy (DSC/TGA/IR), Hot-stage microscopy (HSM) and high-resolution mass spectrometry (HRMS) on samples submitted to water bath at mild temperatures to evaluate a broad spectrum of hazardous compounds formed in the degradation of this product. TGA/IR has revealed that there is effective decomposition in form of CO_2_ along with the formation of hydrogen chloride and other minor compounds. HSM results have provided accurate information, where the melting of the crystals was observed, followed by decomposition. Chlorinated derivatives, including polychlorinated aromatic hydrocarbons (PCAHs) were also confirmed by HRMS. These findings not only corroborate the suspected instability of sucralose to high temperatures, but also indicate that even exposed to mild conditions the formation of hazardous polychlorinated compounds is observed.

Sucralose (1,6-Dichloro-1,6-dideoxy-β-D-fructofuranosyl-4-chloro-4-deoxy-α-D-galactopyranoside) is currently the most utilized artificial sweetener for both industrial purposes and personal use^1^. Although initially considered safe for use^2^, recent literature raised awareness regarding the intrinsic biological effects exhibited by sucralose^3^, as well as the potential that its structure has to hydrolyze into toxic compounds when exposed to severe temperature conditions, forming chloropropanols and other related chlorinated compounds^4^. Important contributions have significantly broadened the knowledge on the accurate conditions under which these undesirable molecules emerge. In 2009, Bannach *et al.* performed thermogravimetry experiments that presented a characteristic decomposition profile for sucralose, indicating that the molecule is unstable (decomposes) in considerably mild temperatures^5^. Further studies utilizing gas chromatography approaches showed that it is possible to assess and monitor the formation of chloropropanols (CPs) in the presence of glycerol^6^, and chlorinated polycyclic compounds in the presence of oils^7^ and metal oxides^8^,^9^. This has intensified the amount of evidence that support the hypothesis that sucralose cannot be suitable for processes that involve temperatures above 120°C up to conditions near pyrolisis.

There is therefore urge to raise questions regarding the safety of non-nutritive sweeteners and bring them to public attention; in past contributions, our group was able to present the formation of potentially health-hazardous byproducts of stevia (*Stevia rebaudiana*) when mixed in low-pH solutions^10^. In this report, we employed a powerful combination of differential scanning calorimetry/thermogravimetric analysis coupled with Fourier transform infrared spectroscopy (DSC/TGA-FTIR), hot-stage microscopy (HSM) and high-resolution mass spectrometry (HRMS) to evaluate the decomposition and elucidate the chemical profile of the compounds that are formed when sucralose is submitted to a relatively mild temperature. Our findings expand the knowledge on chlorinated byproducts, providing strong evidence that the formation of polychlorinated aromatic hydrocarbons (PCAHs) is feasible when isolated sucralose is exposed to temperatures that are even lower than those previously reported in literature.

## Results

### Thermal Analyses

**Figure 1:**
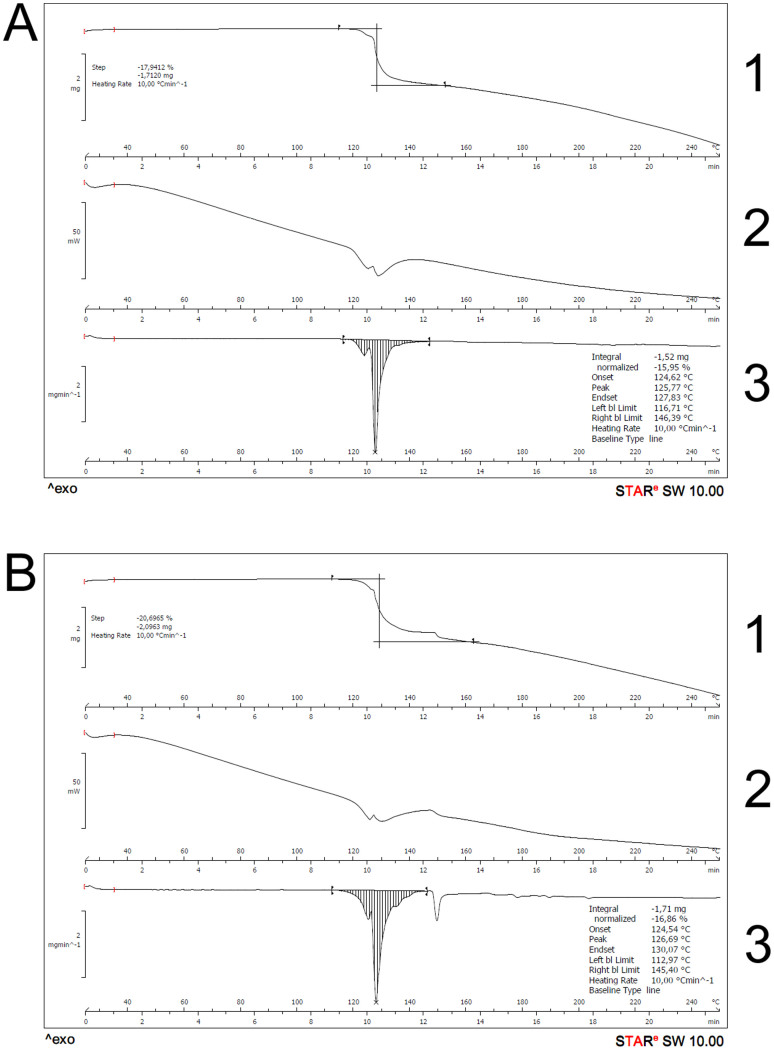
TGA (1 and 3) and DSC (2) curves from both sucralose brands (A and B). It is possible to notice that the mass loss (TGA) and endothermic peak (DSC) happen at the same time during the heat ramp.

**Figure 2:**
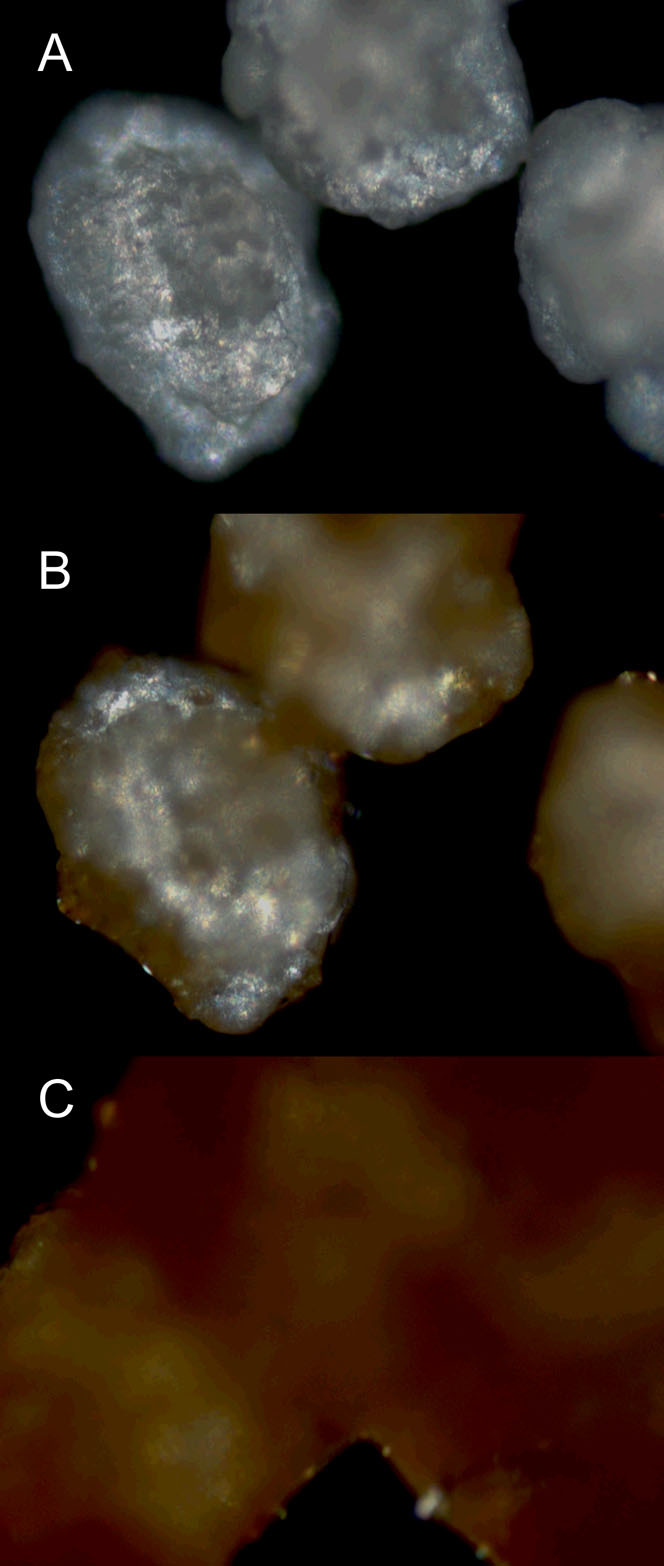
Images of sucralose crystals submitted to HSM analysis. (A) presents the crystals after heating; (B) shows the pre-melting stage and (C) shows the complete melting/caramelization of the crystals.

DSC/TGA results are portrayed in figure 1, where A and B are the two distinct sucralose manufacturers; A1 and B1 present the mass loss observed in the TGA curves, which can be attributable to the loss of several moieties of the parent molecule. It is possible to see that the decomposition of sucralose happens around 125°C (T_onset_ ~ 124.5°C; T_peak_ ~ 125.5°C) for both brands, in a 17–20% rate. Both experiments using N_2_ and synthetic air have presented the same outcome regarding the thermal behavior exhibited, as well as the spectroscopic profile. Figure 2 presents the image profile of sucralose crystals obtained throughout the process of HSM analysis, showing that its crystalline structure is affected at the same temperature range as the one observed in DSC/TGA experiments (around 125°C), in a fusion followed by decomposition process. A time-lapse video depicting the HSM process is available as supplementary material.

### Fourier-transform Infrared Spectroscopy

**Figure 3:**
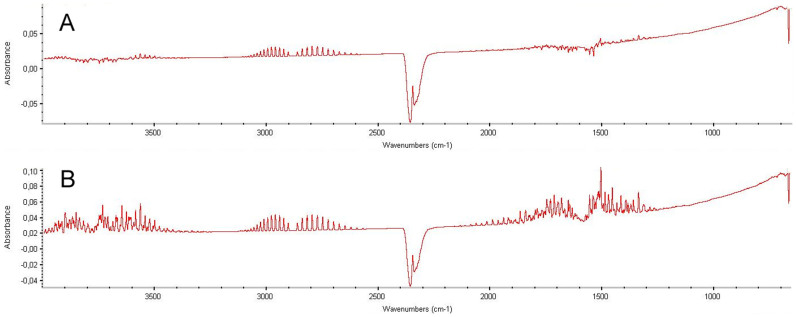
FTIR spectra of both sucralose brands (A and B). Spectral profiles were recorded at the same time (~11 min) for both cases, and present characteristic stretchings for the elucidated compounds.

Sample spectra from coupled FTIR analysis are presented on figure 3. It is possible to notice that both samples from different manufacturers presented the same peak profile (fingerprinting). The depicted spectra are linked with the same runtime where DSC/TGA presented the mass loss curves.

### Mass Spectrometry

**Figure 4:**
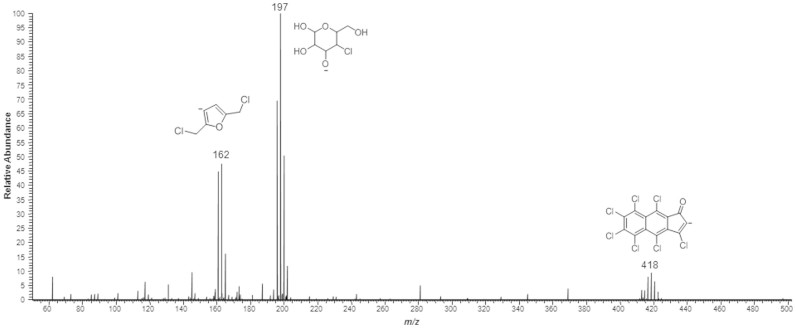
Sample mass spectrum of the air collected from the headspace after heating of sucralose. Identified structures are shown as [M-H]^−^ species. Negative ion mode.

Aromatic chlorinated byproducts were observed in the gaseous phase of the thermal decomposition experiment: a chlorinated furan derivative, similar to hydroxymethylfurfural (HMF) at *m/z* 162, and a compound from the PCAH class at *m/z* 418. Furthermore, a chlorinated tetrahydropyran (equivalent to the glucopyranosyl moiety on the sucrose equivalent) was also observed at *m/z* 197. A spectral sample is available on figure 4, where identified structures are assigned to the observed signals.

## Discussion

DSC curves show an endothermic peak at the same temperature, which may be attributable to the fusion followed by decomposition of the molecule. This is confirmed by HSM data: figure 2 captures the exact transition stage between solid and liquid state. Supplementary data provided present a short movie showing the moment of transition between phases (movie 1). It is possible, therefore, to see that sucralose decomposition occurs right after the fusion of crystals begins, in an almost-simultaneous process. Bannach *et al.* (2009)^5^ have postulated that sucralose decomposes once it reaches the melting point; indeed, in a simple physical state transition there would not be any mass change. Our combination of techniques of thermal analyses with crystal imaging, however, indicates that there is effective melting, even if for a very quick period, prior to decomposition.

FTIR results were processed with software matching using correlation algorithms from spectra libraries. Spectral analysis provide absorbance bands at peaks with characteristic wavenumbers, inferring that, at the decomposition point (linked spectrum at ~11 min), it is possible to observe characteristic profiles of water (ranges from 4000–3200 cm^−1^ and 2000–1200 cm^−1^), carbon dioxide (main peak at 2400–2300 cm^−1^), hydrogen chloride (range from 3100–2600 cm^−1^) and chloroacetaldehyde (main peak at 1850–1700 cm^−1^). This complements the data from DSC/TGA, providing the information that these low molecular weight species must be directly linked to the mass loss events observed in thermal analyses. Furthermore, the observed species are consistent with the expected behavior of a carbohydrate derivative under oxidative conditions (H_2_O and CO_2_ loss), with the addition of chlorinated species due to the presence of such atoms in the molecule.

Spectral analysis of acquired data from HRMS was performed taking into account previous works describing degradation pathways of both sucralose^6^ and sucrose^11^ to guide the search for compounds. Spectral information on figure 4 presents a clear cleavage of the main molecule (chlorinated disaccharide) into two semi-complementary [M-H]^−^ moieties at *m/z* 197 and 162 – the latter being an advanced aromatic degradation product, which follows a hydroxymethylfurfural (HMF)-like pathway from the modified fructose moiety, and the first being a derivative from the modified glucose moiety. Furthermore, at *m/z* 418 it was possible to find a characteristic profile for PCAHs, assigned to a derivative from the complete thermal degradation of sucralose. Interestingly, previous reports have provided information on polychlorinated aromatic species from sucralose^9^, but those experiments were carried out under more sophisticated and particular conditions. Our findings are more closely related to a pyrolytic environment, with molecules developing a mechanism similar to the one that occurs with regular dissacharides, such as sucrose, under these conditions^12^. Since sucralose is a molecule that has three chlorine atoms replacing the usual hydroxyl groups in sucrose, the addition of the halogen may be the key to PCAH formation under considerably mild conditions; there is previous evidence^12^ that carbohydrates that are submitted to harsh temperatures exhibit the tendency to rearrange into thermodynamically stable configurations, resulting in aromatic compounds (either simple or polycyclic). Furthermore, the presence of chlorine increases the potential for reactivity due to an increase in the bond length, especially for the atoms bonded to non-cyclic carbons. These findings indicate, therefore, that potentially hazardous byproducts can effectively emerge even in conditions that can be considered mild, showing that degradation can occur well below the melting point. Despite being a qualitative view, we found strong evidence that PCAHs are formed from sucralose at boiling-water temperatures (up to 98°C), which is the usual temperature reached when preparing hot beverages such as tea or coffee.

This is the first work that reports the thermal behavior of isolated sucralose, with no additional compounds, encompassing a wide range of analytical approaches. Our findings indicate that it is mandatory that the chronic exposure of humans to these chlorinated derivatives is further investigated regarding health-hazardous effects. The use of this artificial sweetener deserves, therefore, close attention, and further research on other food products must be conducted.

## Methods

### Reagents and solvents

Technical-grade sucralose from two distinct manufacturing processes was purchased from local suppliers. Names of the manufacturers remain undisclosed due to legal and ethical issues. Both brands were sampled as follows: 1 g destined to MS analyses and 10 mg for DSC/TGA-FTIR analyses. All utilized solvents were HPLC grade, purchased from J.T. Baker (Xalostoc, Mexico), unless otherwise noticed.

### Thermal analyses

Sucralose samples were introduced into a customized DSC/TGA-FTIR system (DSC-822e/STARe System, Mettler Toledo GmbH, Greifensee, Switzerland; Nicolet™ FTIR module, Thermo Electron Corporation, MA, USA), in a DSC cell previously calibrated using the following high purity metallic standards: indium (T_melt_ = 156.56°C; H_melt_ = 29.13 J.g^−1^) and zinc (T_melt_ = 419.40°C H_melt_ = 109.53 J.g^−1^). Both DSC and TGA curves were performed in a dynamic process, with temperatures ranging from 25°C to 250°C, in a heating program of 10°C/min for scan study. Data acquisition was performed both under inert atmosphere (N_2_) and synthetic air atmosphere, with a flow rate of 50 mL/min in both cases. Samples were deposited in standard aluminum pans with perforated lid, containing 10 mg of sample for DSC/TGA analyses. FTIR results were monitored on-line, and spectra were recorded all along the process, focused on endothermic peaks (DSC) and on the maximum mass variation (TGA). Spectral data were compared to software libraries (HR Aldrich Vapor and HR TGA Vapor Phase) to propose molecular identities. HSM was performed with a hybrid device equipped with a Zeiss Scope. A1 microscope (Carl Zeiss Microscopy, LLC, Thornwood, NY, USA) with polarized light as the image analysis system and a Mettler Toledo FP82HT electrical furnace, controlled by a central processor (FP90, Mettler Toledo). Images were recorded using an AxioVision camera (Carl Zeiss Microscopy, LLC, Thornwood, NY, USA). The heating ramp for HSM was the same as the one utilized for DSC/TGA.

### Mass spectrometry

Samples were placed in a glass vial with a large headspace, capped with a gas-tight lid with silicone septum. The vial was then submerged into a temperature-controlled water bath, where it was allowed to raise temperature at a rate of 1°C/min (from 25°C to 98°C – boiling temperature). After reaching the boiling temperature, the vial remained submerged in the bath for 15 minutes and, after this time, a sample of 10 mL of the air was collected from the headspace using a gas tight syringe (Hamilton Company, Reno, Nevada, USA). The collected air was then dissolved in a 50:50 MeOH/H_2_O solution (1 mL), through the septum of a glass vial. A schematic figure of this experimental setup is available as supplementary material (Figure S1). The final solution was then directly infused into an ESI-LTQ-XL Orbitrap Discovery (Thermo Scientific, Bremen, Germany) high-resolution mass spectrometer (30,000 FWHM). Data acquisition was performed under the following conditions: 4.5 kV spray voltage, 285°C capillary temperature, sheath gas at 10 arbitrary units and a flow rate of 15 mL/min. Negative ion mode was explored, with *m/z* ratios ranging from 50 to 1000. The experiment was conducted in triplicates for both sucralose samples.

### Structure elucidation

High resolution was the parameter of choice for the identification of the chlorinated species in MS analyses. The comparison between theoretical and experimental masses for mass accuracy are given in terms of parts per million (ppm) for error distribution. HRMS (*m/z*): [M]^−^ calcd. for C_6_H_5_Cl_2_O, 162.9723 found, 162.9729 (3.68 ppm). HRMS (*m/z*): [M]^−^ calcd. for C_6_H_10_ClO_5_, 197.0222 found, 197.0227 (2.53 ppm). HRMS (*m/z*): [M]^−^ calcd. for C_13_Cl_7_O, 416.7774 found, 416.7780 (1.43 ppm).

## Author Contributions

D.N.O.: designed and conducted the mass spectrometry experiments, analyzed the results, wrote the paper and prepared the figures. M.M.: designed and conducted the thermal experiments, analyzed the results, wrote the paper and prepared the figures. R.R.C.: idealized all experiments, analyzed the results and revised the paper.

## Supplementary Material

Supplementary Information Supplementary Information

Supplementary Information Supplementary Information
