# Supplementary material for: Thermal degradation of sucralose: a combination of analytical methods to determine stability and chlorinated byproducts
Source: Sci Rep. 2015 Apr 15;5:9598. doi: 10.1038/srep09598 (PMC4397539; doi:10.1038/srep09598)
Supplement: Supplementary Information [file srep09598-s2.pdf]

Supplementary Material from the manuscript: "*Thermal degradation of sucralose: a combination of analytical methods to determine stability and chlorinated byproducts*" by Diogo N. de Oliveira, Maico de Menezes and Rodrigo R. Catharino.

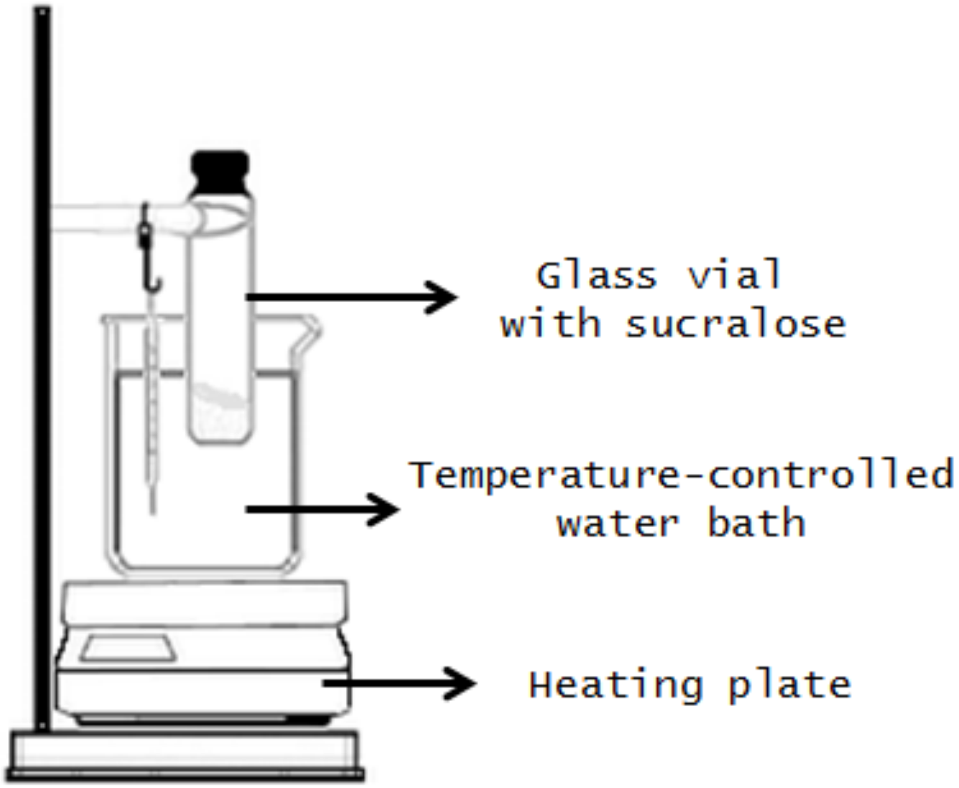

Supplementary Figure 1 (Figure S1). Schematic representation of the experimental setup for the thermal evaluation of sucralose, prior to HRMS analysis.

Supplementary Movie 1. Presents a short motion picture in high resolution of the meltdown process of sucralose using HSM.
